# Clinical Study on the Multidimensional Efficacy of Mindfulness‐Based Stress Reduction Combined With Ear Triple Therapy in Young and Middle‐Aged Patients With Hypertension

**DOI:** 10.1155/ijhy/1020460

**Published:** 2026-06-22

**Authors:** Jing Li, Qingxia Sun, Yanhong Wu

**Affiliations:** ^1^ Affiliated Hospital of Nanjing University of Chinese Medicine (Jiangsu Province Hospital of Chinese Medicine), Nanjing, Jiangsu, China

**Keywords:** ear triple therapy, mindfulness-based stress reduction, patients with hypertension

## Abstract

**Objective:**

This study aims to explore the clinical efficacy of mindfulness‐based stress reduction (MBSR) combined with ear triple therapy in improving blood pressure, symptoms, emotions, and other multidimensional conditions of young and middle‐aged patients with hypertension.

**Methods:**

Using convenient sampling, young and middle‐aged patients with hypertension who visited Jiangsu Provincial Hospital of Chinese Medicine from January 2024 to January 2025 were selected as participants. They were divided into an intervention group and a control group using a random number table, with 115 cases in each group. The control group received standard treatment and nursing, while the intervention group was additionally given MBSR combined with ear triple therapy on the basis of the control group’s intervention, with an intervention cycle of 8 weeks. Before the intervention and after 8 weeks, the therapeutic effect, immediate blood pressure–lowering effect, improvement of TCM symptoms, psychological status, and mindfulness level were compared between the two groups.

**Results:**

There were no significant differences in baseline characteristics and medication use between the 2 groups. After 8 weeks, the intervention group showed superior performance to the control group in terms of therapeutic effect, immediate blood pressure–lowering effect, TCM syndrome score, SAS score, and MAAS score, with statistically significant differences (*p* < 0.05).

**Conclusion:**

MBSR combined with ear triple therapy can improve hypertension conditions in young and middle‐aged patients with hypertension, alleviate symptoms, reduce negative emotions, and enhance mindfulness level, thereby improving medication compliance and self‐management ability.

## 1. Introduction

With the development of society and changes in lifestyle, the incidence of hypertension has been increasing year by year, especially among young and middle‐aged people [1]. Hypertension is a crucial risk factor for cardiovascular diseases, and it can severely affect the prognosis when complicated with target organ damage [2]. Most hypertension cases in young and middle‐aged people are essential hypertension. Studies have shown that [3] poor medication compliance, unhealthy lifestyles, insufficient understanding of the disease, and high life pressure are the main factors leading to poor hypertension control in this population. Long‐term medication, prolonged disease course, and the need for frequent reexaminations can further aggravate patients’ negative emotions such as somatization, obsession, and anxiety, affecting their physical and mental health and leading to various degrees of psychological problems [4]. Mindfulness‐based stress reduction (MBSR) can effectively relieve negative emotional states such as anxiety and tension [5], improve mindfulness level, optimize subjective emotional experience, and help patients manage their emotions and stress effectively, thereby reducing blood pressure elevation caused by negative emotions. Ear triple therapy [6] refers to a therapeutic method that combines three TCM characteristic therapies: ear massage, ear‐tip bloodletting, and auricular point pressing with beans. By stimulating auricular points, it can regulate the Qi and blood throughout the body and alleviate symptoms such as headache and dizziness. However, there are currently no high‐quality clinical research reports on the use of ear triple therapy for hypertension, and some patients also have doubts and fears about this TCM technique. Therefore, this study combined MBSR with ear triple therapy to intervene in both psychological and physiological aspects, aiming to explore their synergistic therapeutic effects, observe their multidimensional impacts on patients’ blood pressure control, TCM syndromes (such as headache and dizziness), psychological status improvement, and mindfulness level enhancement, and provide new ideas for clinical intervention measures for young and middle‐aged patients with hypertension.

## 2. Methods

### 2.1. Ethical Considerations

This study was approved by the Human Research Ethics Committee of the Jiangsu Provincial Hospital of Chinese Medicine (2025NL‐046‐01). All participants provided formal written informed consent, which the committee approved. All names were replaced with codes during electronic data entry to preserve the anonymity and privacy of the participants. The questionnaires were locked in a filing cabinet at the trial hospital. The data were analyzed in an Excel spreadsheet and stored in a password‐protected secure office computer of a researcher. Data security was guarded by firewalls and other security mechanisms imposed by the networked computers at the trial hospital.

### 2.2. Participants and Recruitment

Participants were recruited from January 2024 to January 2025 at Jiangsu Provincial Hospital of Chinese Medicine, Nanjing, China. Inclusion criteria were (1) hypertensive patients aged 35–54 years [7]; (2) patients who met the diagnostic criteria for essential hypertension [8]; and (3) patients with a stable condition, no mental disorders, ability to communicate independently, and willingness to cooperate with the survey. Exclusion criteria were (1) patients with severe impairment of major organ functions; (2) patients with missing ears, obvious ear infections, or skin damage on the ears; (3) patients who requested to withdraw midway; (4) patients with incomplete clinical data; and (5) women in menstruation and patients with coagulation disorders.

Based on the results of previous literature, the total sample size was calculated to be 200 cases. Considering a 15% loss‐to‐follow‐up rate, the final sample size was determined to be 230 cases. The patients were randomized 1:1 to intervention and control groups based on a computer‐generated series of numbers using Excel’s random macro function and a sequentially numbered, sealed envelope technique.

### 2.3. Intervention

The control group received standard treatment and nursing. For treatment, the Expert Consensus on the Management of Young and Middle‐Aged Patients with Hypertension in China [9] was referred to; for patients with Grade 1 hypertension, lifestyle intervention was conducted for several weeks; and if blood pressure still did not reach the target, antihypertensive drug treatment was initiated. For patients with Grade 2 or 3 hypertension and those at high risk of cardiovascular disease, antihypertensive drug treatment was initiated immediately. All patients were informed of disease‐related knowledge, guided to take medications regularly as prescribed and avoid self‐discontinuation of drugs, and simultaneously provided with dietary guidance, daily exercise guidance, and psychological guidance.

The intervention group needs to receive additional MBSR and ear triple therapy besides standard treatment. (1) Ear massage: The patient was assisted to take a comfortable position. The ears were disinfected with 75% alcohol cotton balls, and massage oil was evenly applied to it. Circular massage was performed using the “Small Circulation Method,” starting from auricular points such as Neifenmi, the auricular pituitary point, thyroid reflex area, and auricular thymus reflex area, and then returning to Neifenmi. The “Large Circulation Method” involved circular massage from the great auricular nerve point to the lesser occipital nerve point and the sympathetic point outside the ear tip, then back to the great auricular nerve point. A gentle massage was conducted in the order of the earlobe, helix, tail of helix, upper horn of helix, and protrusion of the triangular fossa. Among them, emphasis was placed on kneading points such as subcortex, Shen, and Pangguang; the anterior and posterior lymph nodes were scraped from top to bottom. The massage time for each ear was controlled at 5–10 min, with the intensity adjusted to the patient’s comfort level. (2) Ear‐tip bloodletting: After repeatedly disinfecting the auricle skin with 75% alcohol cotton balls, a sterile triangular needle was used to quickly prick the ear tip 1‐2 times, and 10–20 drops of blood (each drop approximately the size of a soybean) were released. Finally, sterile cotton balls were used to press and stop bleeding, and the treatment was alternated between the two ears. (3) Auricular point pressing with beans: The auricle was disinfected twice with 75% alcohol cotton balls. The main points selected were auricular blood pressure–lowering groove, Shenmen, Xin, Gan, Shen, and Pixia; auxiliary points were added according to symptoms (e.g., “Zhen” for dizziness, “Temporal Region” for headache, “Sympathetic” for insomnia, and “Fei” for chest tightness). Tweezers were used to take adhesive tape with *Semen vaccariae* (Wangbuliuxing seed) and attach it to the selected auricular points, then gently pressed for 1‐2 min until the patient felt a “Deqi” sensation, a characteristic response in acupuncture referring to soreness, numbness, distension, or heaviness at the acupoint. Patients were instructed to press the points by themselves 3‐4 times a day, 30 s to 1 min per point each time, with the intensity based on tolerance to avoid skin damage caused by excessive pressing. The adhesive tape was retained for 7 days, and the treatment was alternated between the two ears. Ear triple therapy was administered once a week for a total of 8 weeks.

MBSR comprises (1) popularization of basic mindfulness knowledge: Using 8 core dimensions as the framework, patients were systematically taught the core concepts of MBSR and guided to understand its core principle of “being aware of the present moment and accepting oneself,” laying a cognitive foundation for subsequent practice. (2) Disease cognition and body awareness training: First, health education on hypertension was conducted, including disease mechanism, symptom identification, and key points of daily care, to help patients correctly understand their condition. Meanwhile, the method, mechanism, and precautions of ear triple therapy were explained to eliminate patients’ doubts and fears about TCM techniques. Then, patients were guided to conduct body scan exercises: Through progressive body part awareness (from head to feet), patients were led to accurately perceive physical sensations, identify potential physiological symptoms (such as chest tightness and tightness in the shoulders and back), and establish an awareness of the connection between “body signals and disease status.” (3) Core mindfulness skill training: including mindfulness breathing training (based on abdominal breathing, focusing on the natural rhythm of breathing) and meditation practice (cultivating awareness through guided imagery or focusing on specific objects). Patients were guided to establish the cognition that “thoughts are not facts,” helping them learn to distinguish between objective facts, subjective feelings, and automatic thoughts. When facing negative emotions, they could consciously sort out the differences between the three to avoid being affected by negative emotions.

Since patients visited the Hypertension Specialist Clinic, the researchers recorded the contact information of all subjects at enrollment and added the intervention group patients to WeChat. On the day of the medical appointment, the first combined intervention was conducted for the intervention group, and the next intervention time was scheduled. WeChat was used to answer patients’ questions in a timely manner and send reminders about the time and location of the next intervention in advance. The control group received a telephone follow‐up every 2 weeks to reduce subject loss. At the 8th week, patients were notified to be reexamined in the hospital and fill out relevant scales. MBSR was led by 2 nurses with psychological counselor qualifications to ensure the standardization of the process. Ear triple therapy was performed by nurses who had obtained the TCM Nursing Therapist qualification issued by the hospital, ensuring the safety and standardization of the operation. MBSR combined with ear triple therapy was uniformly conducted in the outpatient classroom of the hospital, implemented by 2 qualified TCM nursing therapists and 2 nurses with psychological counselor qualifications to ensure standardized operation. The intervention was performed once a week for 8 weeks, with each session lasting about 60 min.

### 2.4. Evaluation Indicators and Tools

Both the intervention group and the control group received an 8‐week intervention. The following indicators were evaluated before the intervention and after 8 weeks.

#### 2.4.1. Therapeutic Effect

The evaluation criteria were formulated with reference to the Guiding Principles on Clinical Research of New Chinese Medicines [10]. Markedly effective: After treatment, diastolic blood pressure (DBP) decreased by ≥ 10 mmHg and returned to the normal range; or DBP did not return to normal but decreased by ≥ 20 mmHg (meeting either of the two conditions). Effective: After treatment, DBP decreased by < 10 mmHg but reached the normal range; DBP decreased by 10–19 mmHg compared with before treatment but did not reach the normal range; or systolic blood pressure (SBP) decreased by > 30 mmHg compared with before treatment (meeting any of the three conditions). Ineffective: failure to meet the above criteria. Total effective rate = [(number of markedly effective cases + number of effective cases)/total number of observed cases] × 100%.

#### 2.4.2. Immediate Blood Pressure–Lowering Effect

A medical electronic sphygmomanometer was used to measure the blood pressure of both groups at 30, 60, and 120 min after the first treatment.

#### 2.4.3. Improvement of TCM Symptoms

The TCM Symptom Score Scale for Hypertension was used [11]. This scale included 9 items: 3 items were scored on a 0–6 scale, and 6 items on a 0–3 scale, with a total score of 32 points. A lower score indicated milder symptoms.

#### 2.4.4. Comparison of Psychological Status

The Chinese version of the Zung Self‐Rating Anxiety Scale (SAS) was used [12]. It had good reliability and validity and could be used as an effective assessment tool for adult anxiety screening. It included 20 items, with scores ranging from 1 to 4 (from “none or little of the time” to “most or all of the time”). After reverse scoring of the data, the scores of each item were summed up. A higher score indicated a more severe anxiety level.

#### 2.4.5. Comparison of Mindfulness Level

The Mindful Attention Awareness Scale (MAAS), localized by Siyi Chen et al. [13], was used to evaluate the individual mindfulness level. It was a single‐dimensional scale with 15 items, scored from 1 to 6 (from “*almost always*” to “*almost never*”), with a total score ranging from 15 to 90 points. A higher total score indicated a higher mindfulness level.

### 2.5. Statistical Analyses

All data were analyzed using SPSS (Version 21.0; IBM Corp). Measurement data conforming to normal distribution were expressed as mean ± SD, and independent samples *t*‐test and repeated‐measures analysis of variance were used for intergroup comparison. Count data were expressed as frequency and percentage, and a chi‐square test was used for intergroup comparison. A *p* value of < 0.05 was considered statistically significant.

## 3. Results

### 3.1. Participant Characteristics

During the intervention, 5 cases were lost to follow‐up in the intervention group and 3 cases in the control group, with a total of 222 patients completing the study. There were no statistically significant differences in the baseline general information between the two groups (Table 1).

**TABLE 1:** Baseline participant characteristics.

Characteristics		Control group	Intervention group	*χ* ^2^/*t*	*P*
Gender (*n*)	Male	57	58	0.075	0.784
	Female	55	52		

Age (years)		45.33 ± 3.44	44.65 ± 3.38	1.463	0.145

BMI (kg/m^2^)		22.49 ± 1.09	22.17 ± 1.48	1.469	0.118

Blood pressure (mmHg)	SBP	165.59 ± 5.11	163.21 ± 5.82	0.654	0.514
	DBP	100.03 ± 3.15	98.87 ± 3.27	0.125	0.900

Hypertension grade (*n*)	Grade I	17	19	0.473	0.789
	Grade II	87	95		
	Grade III	8	6		

Hypertension course (years)		3.09 ± 1.57	3.11 ± 1.61	0.092	0.926

Family history of hypertension (*n*)		59	64	0.680	0.410

Smoking history (*n*)		55	59	0.456	0.500

Drinking history (*n*)		26	32	0.993	0.319

### 3.2. Therapeutic Effects

The total effective rate of the intervention group was 82.72%, which was significantly higher than that of the control group (67.85%), with a statistically significant difference (*p* < 0.05) (Table 2).

**TABLE 2:** Comparison of clinical therapeutic effects (*n*).

	*n*	Markedly effective	Effective	Ineffective	Total effective rate (%)
Intervention group	110	3	88	19	82.72
Control group	112	1	75	36	67.85
*χ* ^2^	6.601				
*P*	0.037				

### 3.3. Immediate Blood Pressure–Lowering Effect

At 30, 60, and 120 min after treatment, the SBP and DBP of the intervention group were significantly lower than those of the control group (*p* < 0.05) (Tables 3 and 4).

**TABLE 3:** Comparison of SBP (*x* ± *s*, mmHg).

	*n*	SBP		
		30 min	60 min	120 min
Intervention group	110	151.65 ± 5.96	146.67 ± 5.85	139.21 ± 5.55
Control group	112	163.66 ± 5.19	158.55 ± 5.31	149.97 ± 5.14
*t*		10.007	15.850	13.775
*P*		0.001	0.001	0.001

**TABLE 4:** Comparison of DBP (*x* ± *s*, mmHg).

	*n*	DBP		
		30 min	60 min	120 min
Intervention group	110	95.97 ± 3.30	92.02 ± 3.37	88.44 ± 3.28
Control group	112	97.94 ± 3.11	94.88 ± 3.14	91.87 ± 3.07
*t*		4.563	6.516	8.047
*P*		0.001	0.001	0.001

### 3.4. TCM Symptoms

Before the intervention, there was no statistically significant difference in the TCM syndrome score between the two groups. After 8 weeks, the score of the intervention group was lower than that of the control group, with a statistically significant difference (*p* < 0.05) (Table 5).

**TABLE 5:** Comparison of TCM syndrome scores.

	*n*	Baseline	After 8 weeks
Intervention group	110	18.57 ± 2.39	7.47 ± 2.01
Control group	112	17.95 ± 2.74	9.06 ± 1.66
*t*		1.813	6.431
*P*		0.071	0.001

### 3.5. Chinese Version of Zung SAS Scores

Before the intervention, there was no statistically significant difference in the SAS score between the two groups. After 8 weeks, the score of the intervention group was lower than that of the control group, with a statistically significant difference (*p* < 0.05) (Table 6).

**TABLE 6:** Comparison of SAS scores.

	*n*	Baseline	After 8 weeks
Intervention group	110	59.93 ± 2.24	39.5 ± 2.49
Control group	112	60.31 ± 2.71	48.29 ± 2.77
*t*		1.142	24.848
*P*		0.255	0.001

### 3.6. MAAS Scores

Before the intervention, there was no statistically significant difference in the MAAS score between the two groups. After 8 weeks, the score of the intervention group was higher than that of the control group, with a statistically significant difference (*p* < 0.05) (Table 7).

**TABLE 7:** Comparison of MAAS scores.

	*n*	Baseline	After 8 weeks
Intervention group	110	52.37 ± 6.12	72.13 ± 6.46
Control group	112	50.84 ± 9.28	53.30 ± 5.37
*t*		1.451	23.544
*P*		0.148	0.001

## 4. Discussion

### 4.1. MBSR Combined With Ear Triple Therapy Can Significantly Improve Blood Pressure in Young and Middle‐Aged Patients With Hypertension

Our data showed that the total effective rate of the intervention group was 82.72% (3 markedly effective cases, 88 effective cases, and 19 ineffective cases), while that of the control group was 67.85% (1 markedly effective case, 75 effective cases, and 36 ineffective cases). The comparison of therapeutic effects between the two groups after 8 weeks of treatment showed a statistically significant difference (*p* = 0.037 < 0.05), indicating that MBSR combined with ear triple therapy is more effective than conventional treatment and nursing in lowering blood pressure. Shen Juan et al. [14] intervened in hypertensive patients in the cardiology department using triple TCM nursing techniques (auricular point sticking, Chinese herbal foot bath, and Chinese herbal pillow), and the efficacy index of their study was similar to that of this study. In addition, our study found that the SBP and DBP of the intervention group at 30, 60, and 120 min after the first treatment were lower than those of the control group, indicating that MBSR combined with ear triple therapy also has a good immediate blood pressure–lowering effect. According to TCM theory, the etiology and pathogenesis of young and middle‐aged patients with hypertension are mainly “hyperactivity of Gan‐Yang” and “deficiency of Shen‐Yin.” As stated in Huang Di Nei Jing: “The ear is the convergence of the major meridians.” Auricular points are directly connected to Zang‐Fu organs and meridians and collaterals. By stimulating auricular points, Qi and blood throughout the body can be regulated, and dizziness symptoms can be alleviated. Our study selected the main points (antihypertensive groove, Shenmen, Xin, Gan, Shen, and subcortex) that can regulate vascular contraction and relaxation functions, as well as liver and kidney functions. For hypertensive emergencies, additional ear‐tip bloodletting can quickly purge heat and calm Gan‐Yang, achieving an immediate blood pressure–lowering effect [15]. Studies have shown that [16] the auricular concha area is the only superficial area in the human body with vagus nerve afferent fiber distribution. Ear triple therapy can stimulate the auricular vagus nerve, balance the sympathetic–parasympathetic nervous system, and assist in lowering blood pressure. With the transformation of the medical model to the biopsychosocial model, the impact of negative emotions on diseases has received increasing attention. Studies have shown that [17] hypertensive patients are closely associated with negative emotions such as anxiety and depression. A meta‐analysis [18] showed that anxiety can affect sympathetic nerve function, leading to an imbalance in the release of vasoconstrictor and vasodilator substances from vascular endothelium, thereby affecting blood pressure. MBSR is a mindfulness‐based stress management method. Through mindfulness meditation and other training, it reduces the subject’s stress and enables practitioners to continuously focus on and be aware of the current experience with an accepting and nonjudgmental attitude [19]. Mindfulness can directly stimulate cardiopulmonary receptors and affect the cardiovascular central nervous system and the activity of the sympathetic–vagus nervous system through interactions between central systems, thereby regulating blood pressure [5]. Studies have shown that MBSR can improve the healthy lifestyle of hypertensive patients and reduce their blood pressure levels [20]. MBSR combined with ear triple therapy can achieve dual psychological–physiological regulation, synergistically lower blood pressure, improve patients’ intervention compliance and self‐management ability, reduce drug dependence, and achieve a better blood pressure–lowering effect.

### 4.2. MBSR Combined With Ear Triple Therapy Can Significantly Reduce TCM Syndrome Scores in Young and Middle‐Aged Patients With Hypertension

The TCM syndrome scores for hypertension in young and middle‐aged patients mainly include dizziness, headache, irritability, facial flushing, conjunctival hyperemia, xerostomia, bitter taste in the mouth, constipation, and hematuria. The results of this study showed that the TCM syndrome score in the intervention group decreased significantly after intervention, which was better than that before the intervention; the difference was statistically significant compared with the control group (*p* < 0.05). This suggests that MBSR combined with ear triple therapy can effectively alleviate hypertension‐related symptoms. Ear triple therapy includes ear massage, ear‐tip bloodletting, and auricular point pressing with beans. Ear massage stimulates acupoints on the auricle to regulate the circulation of Qi and blood throughout the body, achieving the effects of dredging meridians, promoting blood circulation to remove blood stasis, reducing swelling, and relieving pain. The ear‐tip point is an extra‐meridian point with the effects of improving eyesight, lowering blood pressure, and detoxifying. Ear‐tip bloodletting can expel pathogenic heat outward, achieving the effects of calming Gan‐Yang and refreshing the mind. Auricular point pressing with beans adopts the “purgation method”: By pressing Wang‐Bu‐Liu‐Xing seed on the corresponding auricular points, it can dredge meridians, regulate Qi and blood, and restore the balance of Yin and Yang. Ren et al. [21] applied ear triple therapy to patients with dizziness due to hyperactivity of Gan‐Yang, and the study showed that it could effectively alleviate symptoms, regulate anxiety status, improve quality of life and therapeutic efficacy, and reduce the recurrence rate. Xu et al. [22] found that ear triple therapy in the treatment of essential headache due to Qi stagnation and blood stasis can improve the degree of headache and psychological status. MBSR can improve patients’ cognition and thinking patterns, help the body achieve a relaxed state, and enhance patients’ self‐control ability and positive emotions, thereby alleviating irritability. Ear triple therapy can directly stimulate Zang‐Fu organs and meridians to alleviate physical symptoms and reduce TCM syndrome manifestations; MBSR improves emotional factors to block the vicious cycle of “negative emotions and aggravated TCM syndromes.” The combination of the two therapies can significantly reduce TCM syndrome scores.

### 4.3. MBSR Combined With Ear Triple Therapy Can Improve Mindfulness Level and Alleviate Anxiety in Young and Middle‐Aged Patients With Hypertension

Young and middle‐aged patients face multiple pressures from family, career, and society. In addition, factors such as mental tension, dietary disorders, imbalance between work and rest, and congenital endowment lead to disorders of liver and spleen functions, such as phlegm dampness due to spleen deficiency or hyperactivity of Yang caused by Gan‐Qi stagnation [23]. The results showed that after intervention, the SAS scores and MAAS scores of both groups were better than those before the intervention, and the intervention group was superior to the control group, with statistically significant differences. Yang et al. [24] intervened in patients with coronary heart disease complicated by hypertension using MBSR, and the preintervention SAS scores were similar to those in this study, while the postintervention results of this study were better. The analysis suggests that our study participants were young and middle‐aged, who had better comprehension, compliance, and adjustment ability than the elderly group. Through a progressive 8‐week intervention, MBSR helps patients correctly understand the disease, then conducts body scan exercises, and continuously strengthens the practical application ability of mindfulness skills to alleviate anxiety and relax the body and mind. Studies have shown that mindfulness training can effectively reduce patients’ depression and anxiety by activating the emotional and emotion‐regulation brain region: the left prefrontal cortex [25]. In addition, relevant studies have shown that mindfulness‐based psychological nursing can eliminate patients’ uncertainty about the disease, improve posttreatment medical compliance, post–disease safety, and nursing satisfaction [26]. MBSR combined with ear triple therapy works in two aspects: On the one hand, ear treatment provides feedback regulation of the corresponding Zang‐Fu functions, improves hypertension status, reduces symptoms such as dizziness, headache, and irritability, and improves physical and mental discomfort overall; on the other hand, mindfulness concepts are established, and mindfulness breathing training and meditation training are used to improve patients’ mental state and enhance emotion regulation ability. Through disease knowledge and body awareness training, patients are helped to deepen their understanding of their self‐role and face the disease more calmly and peacefully. The synergy of the two further improves blood pressure status and adjusts psychological state.

## 5. Conclusion

Our study shows that MBSR combined with ear triple therapy can significantly improve hypertension in young and middle‐aged patients, which provides certain enlightenment for clinical nursing. Both therapies are nonpharmacological therapies, and their combination achieves a good therapeutic effect while optimizing patients’ psychological state, improving disease cognition, and enhancing patients’ mindfulness level, which can indirectly improve medication compliance and self‐management ability. Future studies can expand sample size, conduct multicenter studies, extend the intervention cycle, and comprehensively evaluate the efficacy of MBSR combined with ear triple therapy, aiming to provide more diversified medical options besides drug treatment for middle‐aged and young hypertensive patients.

## Funding

This work was supported by the Hospital‐Level Project of Affiliated Hospital of Nanjing University of Chinese Medicine (Grant Number [Y24071]).

## Ethics Statement

This study was approved by the Human Research Ethics Committee of the Jiangsu Provincial Hospital of Chinese Medicine (2025NL‐046‐01). All participants provided formal written informed consent, which the committee approved.

## Conflicts of Interest

The authors declare no conflicts of interest.

## Data Availability

The datasets generated and/or analyzed during the current study are not publicly available because the data are part of an unpublished dissertation, but are available from the corresponding author upon reasonable request.
